# Reactive Blending of Recycled Poly(ethylene terephthalate)/Recycled Polypropylene: Kinetics Modeling of Non-Isothermal Crystallization

**DOI:** 10.1021/acsomega.2c08027

**Published:** 2023-04-18

**Authors:** Aboulfazl Barati, Pixiang Wang, Shaoyang Liu, Erfan Dashtimoghadam

**Affiliations:** Center for Materials and Manufacturing Sciences, Departments of Chemistry and Physics, Troy University, Troy, Alabama 36082, United States

## Abstract

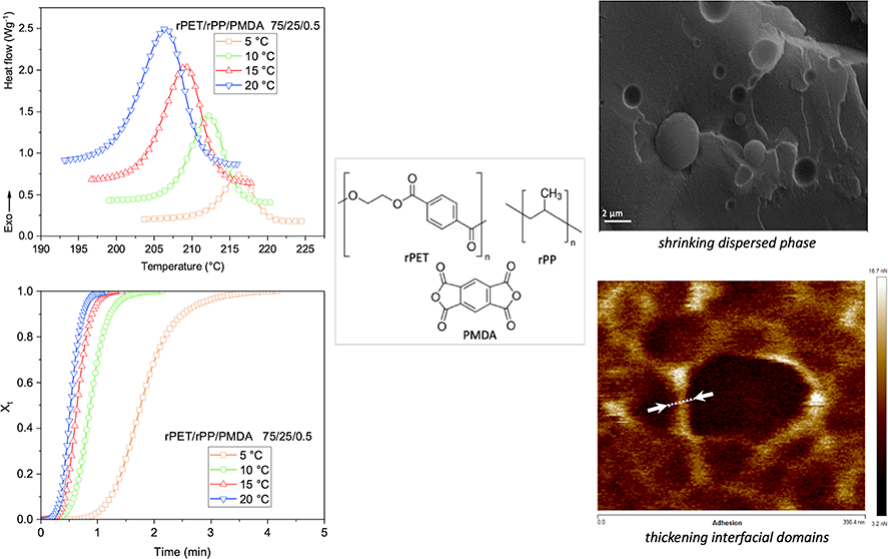

Plastics were developed to change our world for the better. However, plastic pollution has become a serious global environmental crisis. Thermoplastic polyesters and polyolefins are among the most abundant plastic waste. This work presents an in-depth non-isothermal crystallization kinetics analysis of recycled post-consumer poly(ethylene terephthalate) (rPET) and recycled polypropylene (rPP) blends prepared through reactive compounding. The effect of pyromellitic dianhydride (PMDA) on crystallization kinetics and phase morphology of rPET/rPP blends was investigated by differential scanning calorimetry (DSC) and microscopy techniques. DSC results showed that increasing rPP content accelerated rPET crystallization while reducing crystallinity, which indicates the nucleation effect of the rPP phase in blends. Further, it was found that the incorporation of PMDA increased the degree of crystallinity during non-isothermal crystallization, even though the rate of crystallinity decreased slightly due to its restriction effects. The non-isothermal crystallization kinetics was analyzed based on the theoretical models developed by Jeziorny, Ozawa, Mo, and Tobin. The activation energy of the crystallization process derived from Kissinger, Takhor, and Augis–Bennett models was found to increase in rPET/rPP blends with increasing PMDA due to hindered dynamics of the system. Rheological measurements revealed that rPET melt viscosity is remarkably increased in the presence of PMDA and reactive blending with rPP relevant for processing. Moreover, nanomechanical mapping of the rPP phase dispersed in the rPET matrix demonstrated the broadening of the interfacial domains after reactive blending due to the branching effect of PMDA. Findings from this study are essential for the recycling/upcycling thermoplastics through non-isothermal fabrication processes, such as extrusion and injection molding, to mitigate the lack of sorting options.

## 1 Introduction

World population growth, improved living standards, and consequently increasing polymer demand have resulted in evergrowing plastic waste.^1^ The degradation of plastics by microorganisms is complex, unlike decomposition of other forms of biomass (e.g., cellulose). Several environmental problems are caused by large plastic pieces and microplastics, affecting human health and the ecosystem.^2,3^

Poly(ethylene terephthalate) (PET) is a thermoplastic polyester widely used in textiles, soft-drink bottles, and packaging. PET is the most commonly recycled commodity plastic.^4^ Among polyolefins, polypropylene (PP) is a popular plastic of choice for various applications. However, only about 1% of post-consumer PP waste is recycled. PP has a high melt viscosity, which makes its direct processing difficult through extrusion and injection molding.^5^ Due to increased awareness of environmental issues caused by plastic pollution, researchers have been trying to find efficient and cost-effective solutions for polymer recycling.^6,7^ In contrast to landfill disposal, plastic waste can be recovered materially and energetically.^8^ A recycling process (mechanical, chemical, and feedstock recycling) involves material recovery from plastic waste streams, while an energy recovery procedure involves the combustion of waste to generate heat.^9−11^ Formulation of polymer blends and composites is one way to reuse plastic waste.^12,13^ Because of improved processing, low cost, and decent mechanical properties, blending recycled PET (rPET) with recycled polyolefins (e.g., recycled polyethylene, rPP) has emerged as an attractive approach.^14,15^ PET typically has a solubility parameter (δ) value of about 22 MPa^1/2^, while PP covers a range of 16.16–19.23 MPa^1/2^, implying the immiscibility of polymers.^16,17^ The poor compatibility due to the difference in polarity of rPET and rPP requires addressing the dispersion and interfacial adhesion issue.^18^ This compatibility can be improved using compatibilizers, such as PP grafted with maleic anhydride (PP-*g*-MA),^19^ PP grafted with acrylic acid (PP-*g*-AA),^20^ and ethylene-glycidyl methacrylate (EGMA).^21^

As PET requires processing at high temperatures, a series of thermal and hydrolytic degradation reactions occur in the presence of water and other residues.^22,23^ This cause the formation of shorter PET chains with an increase in carboxyl and hydroxyl end groups. A variety of strategies were used to overcome this problem. Many studies focused on extending, branching, and crosslinking of PET chains.^24−26^ In the chain extension process small molecules with two or more functional groups react with PET carboxyl/hydroxyl functional groups to polymerize oligomers and/or broken segments generated from PET chain scission. Different types of chain extenders by adding reactive functional groups, including trimethyl trimellitate,^27^ isocyanates,^28^ epoxy-based compounds,^29^ bis-oxazolines,^30^ and organic phosphites^31^ are effective at increasing intrinsic viscosity, molecular mass, and melt strength. The effect of pyromellitic dianhydride (PMDA), as the most commonly used chain extender, on the properties of PET has been comprehensively investigated.^32−34^ Incorporation of PMDA significantly decreases the carboxyl content, increases the intrinsic melt viscosity, and improves its rheological behavior. These properties are associated with increased molecular weight (MW) and chain entanglement. According to Incarnato et al., modified PET with PMDA showed increased complex viscosity and melt strength.^35^ By using a co-rotating twin screw extruder, Awaja et al. investigated the effect of PMDA and extrusion residence time on PET thermal properties and crystallization behavior.^36^ Their temperature-controlled differential scanning calorimetry (DSC) results showed no significant change in the glass transition temperature (*T*_g_) with PMDA while the residence time increased. As the residence time and the PMDA content increased, the melting temperature and crystallization temperature of PET decreased. Härth et al. studied the strain hardening and shear thinning of PET in the presence of PMDA. They found that chain extension reactions (tree-like branch-on-branch structure) resulted in increased MW and a broader MW distribution.^37^

In crystalline polymers, crystallization behavior plays an essential role in their mechanical properties. The crystallization behavior is usually investigated using non-isothermal kinetics. The crystallization behavior of PET/PP blends relies on their compatibility and heterogeneous nucleation. To analyze the crystallization kinetics of pristine PP and PET/PP blends, Li et al.^38^ applied the mathematical models developed by Jeziorny, Ozawa, and Mo, which showed PET in situ microfibers significantly nucleated the PP phase. Zhu et al. observed that PET increases the crystallization temperature.^39^ Compatibilization of PP/PET blends using PP-*g*-MA demonstrated a significant effect on the crystallization behavior of PP. The same results were reported by Zhidan et al.^40^ and Tao et al.^41^ using PP-*g*-MA and PP-*g*-GMA (PP grafted with glycidyl methacrylate), respectively.

Crystallization rate is of primary importance in non-isothermal polymer fabrication processes, such as injection molding and extrusion.^42^ In order to understand the non-isothermal crystallization behavior of blends, it is necessary to determine how the blend composition and processing conditions affect this behavior. In this work, the effects of the chain extender (PMDA) and cooling rate on the crystallization behavior of rPET in rPET/rPP/PMDA blends (without using compatibilizers) are investigated by differential scanning calorimetry (DSC). Kinetics models are implemented to study non-isothermal crystallization. The activation energy for crystallization is derived from Kissinger, Takhor, and Augis–Bennett methods. It is envisioned the present detailed study on crystallization effects provide insight into efficient melt processing of post-industrial thermoplastics waste to fit the new applications.

## 2 Results and Discussion

**Figure 1:**
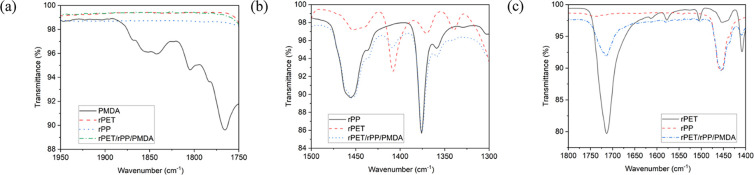
(a–c) Fourier transform infrared spectra of rPET, rPP, PMDA, and rPET/rPP/PMDA blend over different wavenumber ranges.

Fourier transform infrared (FTIR) spectroscopy was used to examine the reaction between rPET and PMDA. The characteristic peaks of PMDA anhydride functionality are located at about 1770 and 1860 cm^–1^ (Figure 1a). As can be seen, these peaks disappeared in the rPET/rPP/PMDA blend, indicating all anhydrides reacted with PET hydroxyl end groups to form star-like branch structures.^43^ Asymmetric and symmetric in-plane C–H at 1455 and a shoulder at 1358 cm^–1^ are the well-documented bands for rPP (Figure 1b). The peak at 1376 cm^–1^ is assigned to the −CH_3_ group. As shown in Figure 1c, rPET shows peaks at 1715 and 1410 cm^–1^ attributed to the stretching vibration of the carbonyl group and the benzyl ring of PET, respectively. The peaks at 1504 and 1577 cm^–1^ are associated with C=C stretching vibrations in the backbone of PET. The spectra revealed rPET and rPP do not undergo chemical interactions.

**Figure 2:**
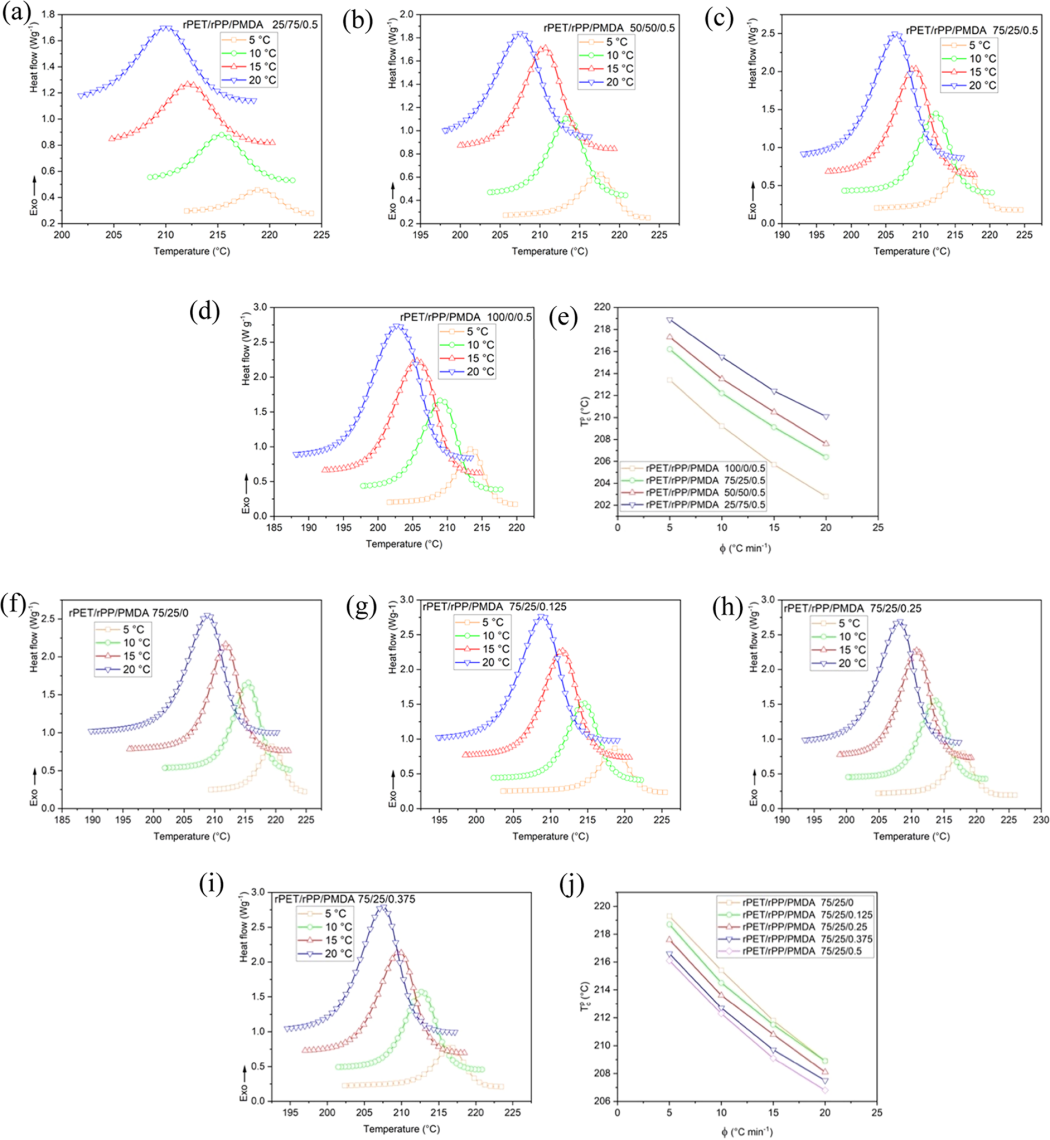
Crystallization exotherms of rPET/rPP/PMDA blends at different rPET/rPP percentages of (a) 25/75, (b) 50/50, (c) 75/25, (d) 100/0, and PMDA 0.5 wt %. (e) Variation of crystallization peak temperature of rPET at different cooling rates and rPET/rPP compositions (samples P1–P4). The crystallization exotherms of the rPET/rPP 75/25 blend at different PMDA wt % of (f) 0 wt %, (g) 0.125 wt %, (h) 0.25 wt %, and (i) 0.375 wt % (and (c) 0.5 wt %). (j) Variation of crystallization peak temperature of rPET in rPET/rPP 75/25 as a function of the cooling rate at different PMDA wt % (samples E1–E5).

Nucleation and growth are two sequential steps in the crystallization process. For the homogeneous melt of a neat polymer, when the temperature is lowered below the melting point (*T*_m_), nucleation starts by spontaneous aggregation of polymer chains due to decreased mobility. In the case of the rPET/rPP blends, a heterogeneous melt is formed and rPP chains act as nucleating domains for rPET crystallization. The crystallization exotherms for rPET and rPET/rPP/PMDA blends with various compositions at different cooling rates of 5, 10, 15, and 20 °C min^–1^ are displayed in Figure 2. With increasing cooling rate, rPET crystallized at lower temperatures in terms of both the onset (*T*_c_^on^) and peak (*T*_c_^p^) temperature. The higher the cooling rate (5–20 °C min^–1^), the shorter it takes for rPET chains to crystallize. However, a high cooling rate (i.e., rapid crystallization) causes the formation of an imperfect crystalline structure. The area under rPET exothermal crystallization peaks measures the crystallinity of its domains. A decrease in Δ*H*_c_ with increasing rPP/rPET ratio indicates rPET crystallization significantly diminished in the presence of rPP chains. At a given cooling rate, *T*_c_^on^ and *T*_c_^p^ of rPET in the blends were found to increase with decreasing rPET/rPP ratio (Figure 2e). This finding is consistent with previous reports, which studied the effect of heterogeneous nucleation on crystallization.^39^ Increasing PMDA was found to lower the *T*_c_^on^ and *T*_c_^p^ (Figure 2j). Similar results for trimethyl trimellitate (TMT) have been reported elsewhere.^44^ This effect was attributed to introduction of long-chain branches (LCBs) into PET. Similarly, PMDA can add up to four branches to its reactive sites. LCBs increase the MW and MW distribution of rPET, while decreasing the chain mobility. This effect is intensified by increasing the PMDA content in blends.

**Figure 3:**
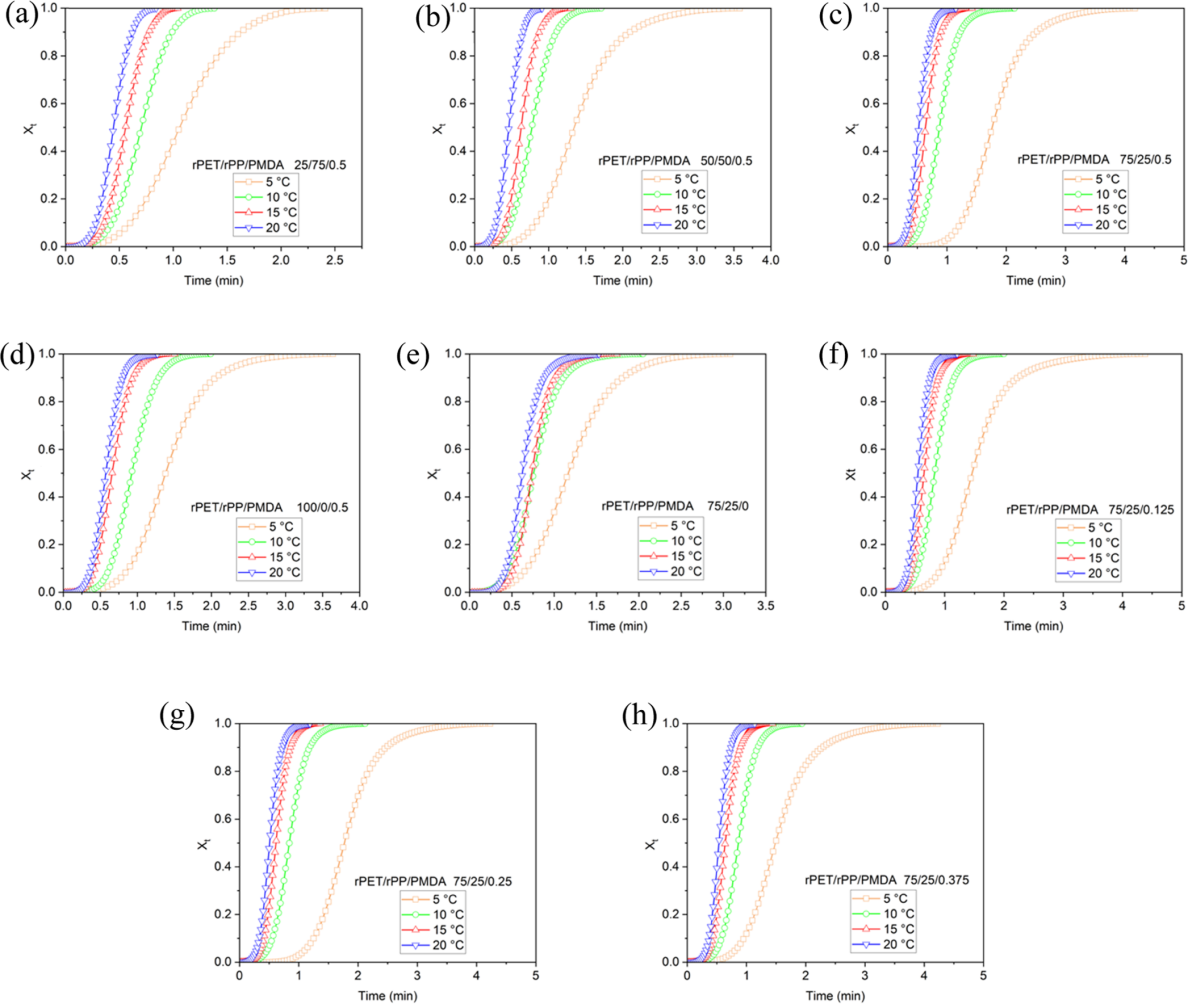
Relative degree of crystallinity (*X*_*t*_) as a function of time for rPET/rPP/PMDA blends at different rPET/rPP composition percentages of (a) 25/75, (b) 50/50, (c) 75/25, (d) 100/0, and PMDA 0.5 wt %. Plots of *X*_*t*_ versus *t* for rPET/rPP 75/25 blends at different PMDA contents of (e) 0 wt %, (f) 0.125 wt %, (g) 0.25 wt %, and (h) 0.375 wt %, (and (c) 0.5 wt %).

The relative degree of crystallinity, *X*_*t*_, as a function of time, *t*, for rPET/rPP/PMDA blends at different compositions and different cooling rates are shown in Figure 3. The sigmoidal shape of curves is associated with a lag between the cooling rate and crystallization time. When the temperature is lowered below the melting point, thermodynamic factors with the lowest and most stable energy may induce chain crystallization. As can be seen, slopes of the *X*_*t*_ curves for a given sample significantly increase as the cooling rate increases. Increasing the rPP phase and the PMDA content in the rPET matrix increased *X*_*t*_ at a given cooling rate. The half-time (*t*_1/2_) of non-isothermal crystallization of rPET in blends based on *t*_0.5_ = (ln 2/*k*)^1/*n*^ are summarized in Table 1.

**Table 1:** Differential Scanning Calorimetry Analysis and Parameters of the Kinetic Model (Avrami, Jeziorny-Modified Avrami, Tobin, and Khanna) for rPET in Blends with rPP/PMDA at Different Cooling Rates^a^

sample	φ (°C min^–1^)	*T*_c_^on^ (°C)	*T*_c_^p^ (°C)	*t*_1/2_ (min)	*n*	*Z*_*t*_	*Z*_c_	*n*_T_	*K*_T_	CRC
P1	5	222.4	218.9	1.07	3.25	0.55	0.89	–4.24	0.87	–1.68
	10	219.5	215.5	0.71	3.83	2.62	1.10	–4.47	5.61	
	15	216.6	212.4	0.56	4.08	7.19	1.14	–5.46	27.16	
	20	214.6	210.1	0.45	3.98	16.72	1.15	–5.34	84.68	
P2	5	220.9	217.3	1.39	3.29	0.23	0.75	–4.69	0.26	–1.55
	10	217.4	213.5	0.79	3.81	1.72	1.05	–5.19	4.15	
	15	214.7	210.5	0.64	4.18	4.39	1.10	–5.93	16.91	
	20	212.3	207.6	0.47	3.87	12.99	1.13	–5.25	62.85	
P3	5	219.8	216.2	1.83	4.09	0.06	0.56	–6.24	0.03	–1.52
	10	216.1	212.2	0.89	3.87	1.05	1.00	–5.74	2.36	
	15	213.2	209.1	0.65	3.95	3.69	1.09	–5.69	13.82	
	20	211.1	206.4	0.54	3.92	7.78	1.11	–5.61	39.48	
P4	5	216.7	213.4	1.43	3.51	0.19	0.72	–5.09	0.21	–1.41
	10	213.2	209.2	0.95	4.17	0.87	0.98	–5.91	1.69	
	15	210.5	205.7	0.67	3.78	3.08	1.08	–5.34	10.09	
	20	208.5	202.8	0.59	3.88	5.48	1.09	–5.51	23.21	
E1	5	222.8	219.3	1.21	4.33	0.39	0.83	–4.33	0.56	–1.43
	10	219.1	215.4	0.78	4.99	1.61	1.04	–4.99	4.33	
	15	215.9	211.8	0.76	6.67	2.13	1.05	–6.67	8.13	
	20	213.6	208.9	0.65	6.15	3.68	1.07	–6.15	18.41	
E2	5	222.1	218.7	1.54	3.21	0.17	0.71	–5.15	0.15	–1.52
	10	218.1	214.5	0.85	3.87	1.28	1.02	–5.67	3.11	
	15	215.4	211.5	0.67	4.18	3.71	1.09	–6.18	15.14	
	20	213.2	208.9	0.57	4.17	7.44	1.11	–6.17	42.51	
E3	5	221.1	217.6	1.84	4.15	0.05	0.56	–6.47	0.02	–1.58
	10	217.2	213.6	0.87	3.78	1.19	1.01	–5.64	2.87	
	15	214.7	210.8	0.63	4.02	4.41	1.10	–5.78	17.86	
	20	212.3	208.1	0.53	3.77	7.87	1.11	–5.53	44.74	
E4	5	220.1	216.6	1.56	3.33	0.16	0.69	–5.08	0.14	–1.62
	10	216.4	212.7	0.88	4.11	1.15	1.01	–5.94	2.59	
	15	213.7	209.7	0.66	3.96	3.63	1.08	–5.76	13.87	
	20	211.6	207.5	0.55	4.21	8.53	1.11	–6.15	49.19	
E5	5	219.7	216.1	1.57	3.73	0.13	0.66	–5.38	0.11	–1.59
	10	215.9	212.3	0.92	3.96	0.99	0.99	–5.86	2.13	
	15	213.2	209.1	0.66	3.91	3.51	1.08	–5.53	12.36	
	20	211.1	206.8	0.55	4.26	9.18	1.12	–6.04	48.06	

a See Table 3 for sample compositions; φ: cooling rate, crystallization onset (*T*_c_^on^), peak (*T*_c_^p^) temperature, *t*_1/2_: non-isothermal crystallization half-time, *n*: Avrami exponent, Avrami (*Z*_*t*_), Jeziorny extended Avrami (*Z*_c_) rate constant, *n*_T_: Tobin exponent, *K*_T_: Tobin rate constant, CRC: crystallization rate coefficient (see Table 4).

For all blend compositions, *t*_1/2_ for rPET was found to decrease with increasing cooling rate (5–20 °C min^–1^).^38^ At a given cooling rate, adding rPP shortened the *t*_1/2_ of the rPET/PMDA blend, indicating that the nucleation effect of rPP enhances the crystallization rate of rPET. It is worth noting that blend samples P1–3 showed shorter *t*_1/2_ than homopolymer sample P4, which implies the rPP heterogeneous nucleation effect.

The crystallization rate can be estimated in terms of the crystallization rate coefficient, CRC = Δφ/Δ*T*_c_^p^, which is inversely proportional to the crystallization rate.^45^ At a given cooling rate, *CRC* results showed a decrease with increasing rPP content (Table 1), which is well consistent with the results presented above (Figure 3). Further, CRC was found to decrease with increasing PMDA content at a given cooling rate, which intensified the restriction effect.

**Figure 4:**
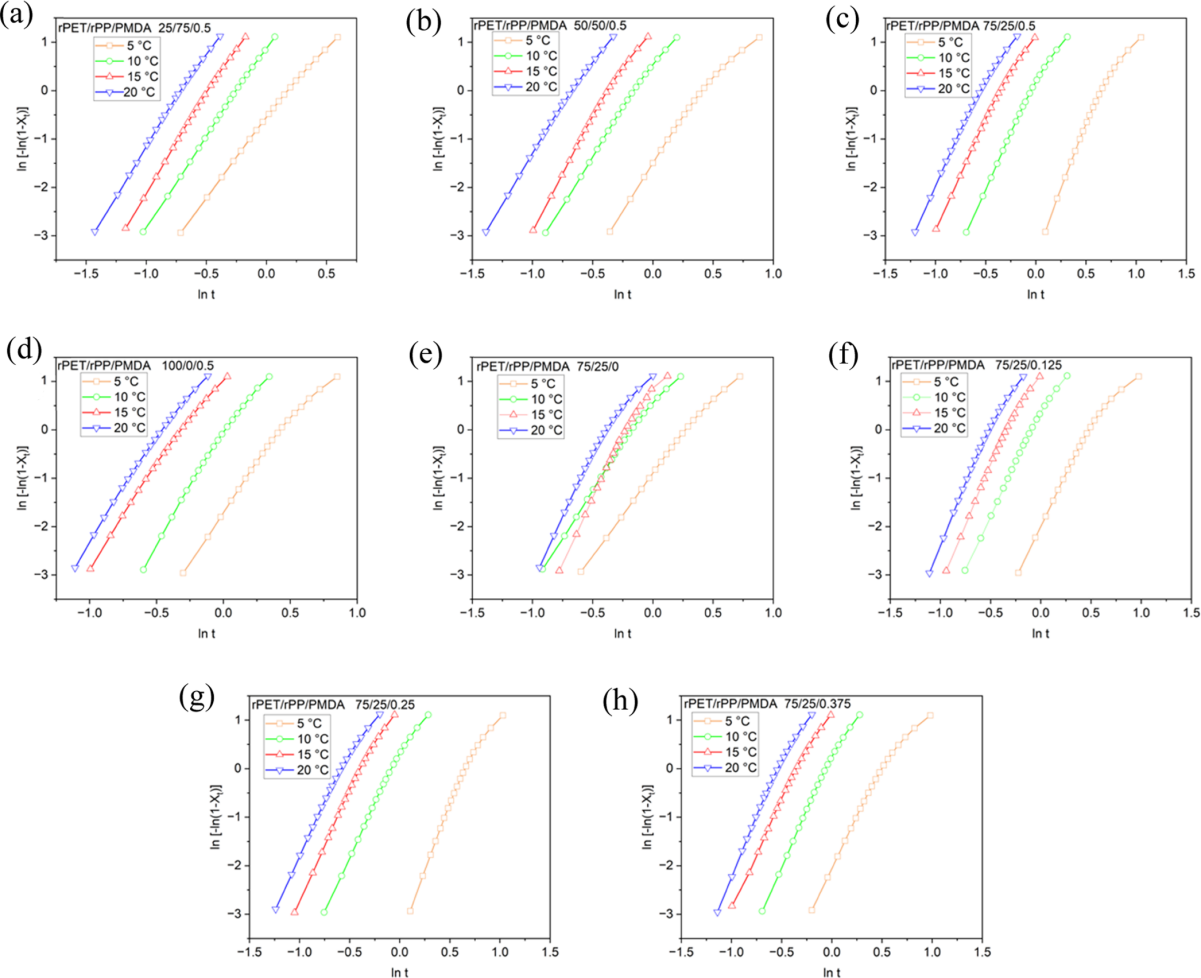
(a) Plots of ln[−ln(1 – *X_t_*)] versus ln *t* (*X*_*t*_: relative degree of crystallinity, *t*: time) for rPET/rPP/PMDA blends at different rPET/rPP content percentage of (a) 25/75, (b) 50/50, (c) 75/25, (d) 100/0, and PMDA 0.5 wt %. Plots of ln[−ln(1 – *X_t_*)] versus *t* for rPET/rPP 75/25 blend at different PMDA contents of (e) 0 wt %, (f) 0.125 wt %, (g) 0.25 wt %, (h) 0.375 wt %, and ((c) 0.5 wt %).

Four kinetics models (Table 1) were adopted in this study to investigate non-isothermal crystallization of the rPET phase in blends with rPP compounded in the presence of PMDA. Figure 4 displays the results of applying Jeziorny modification to the Avrami equation to analyze non-isothermal crystallization. The primary and secondary crystallization stages are distinct in all plots. On each curve, a point of non-linearity separates the two stages. When rPP is added, the crystallinity of the turning point increased. At a cooling rate of 5 °C min^–1^, crystallinity of rPET in sample P4 was ca. 65% at the non-linearity turning point, whereas it was found to be 75, 80, and 85% for P3, P2, and P1 compositions, respectively, which are associated with the nucleation effect of rPP, promoting the primary crystallization of the rPET phase. The crystallinity of rPET at the non-linearity point was increased by increasing PMDA in blends. The primary stage of crystallinity of rPET at this turning point was ca. 50, 55, 61, and 75 for PMDA 0.125, 0.25, 0.375, and 0.5 wt %, respectively.

**Figure 5:**
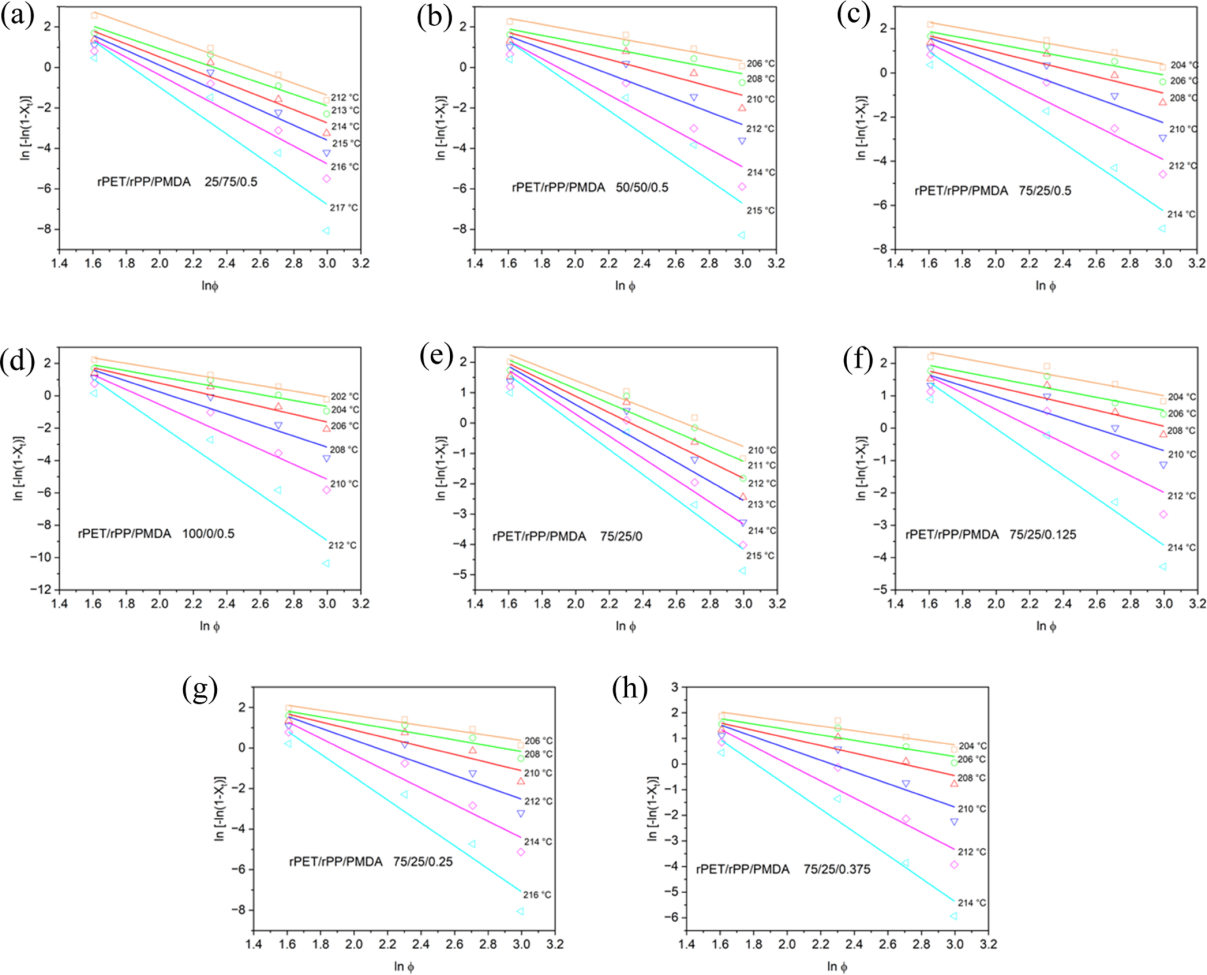
Plots of ln[−ln(1 – *X_t_*)] as a function of ln φ (*X*_*t*_: relative degree of crystallinity at time *t*, φ: cooling rate) for rPET/rPP/PMDA blends at different composition percentages of rPET/rPP (a) 25/75, (b) 50/50, (c) 75/25, (d) 100/0, and PMDA 0.5 wt %. Plots of ln[−ln(1 – *X_t_*)] versus ln φ for rPET/rPP 75/25 blend at different PMDA content of (e) 0, (f) 0.125, (g) 0.25, (h) 0.375, and (and (c) 0.5 wt %).

Figure 5 shows a linear relationship between ln[−ln(1 – *X_t_*)] and ln φ based on the Ozawa model. For rPET blends, the correlation coefficients of linear fitting were low, suggesting that the Ozawa method cannot be adopted for these materials. Similarly, it has been reported elsewhere that the Ozawa method cannot be applied to polymers where crystallization processes differ.^39,46^

**Figure 6:**
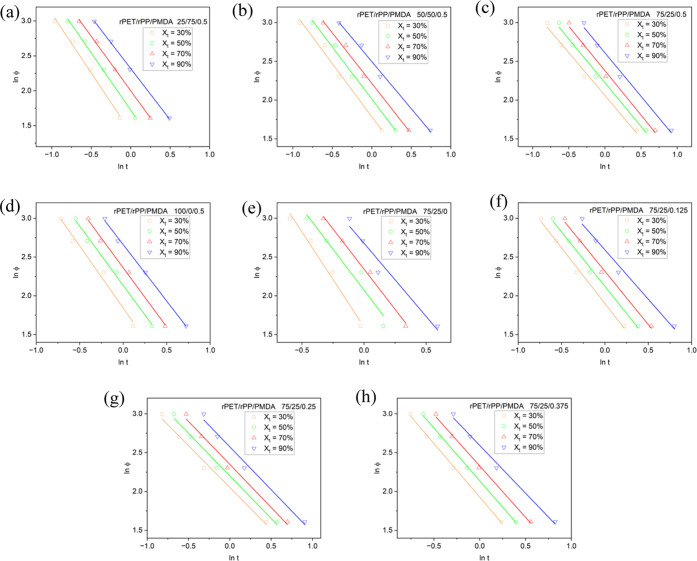
Plots of ln φ versus ln *t* (φ: cooling rate, *t*: time) for rPET/rPP/PMDA blends at different rPET/rPP content percentages of (a) 25/75, (b) 50/50, (c) 75/25, (d) 100/0, and PMDA 0.5 wt %. Plots of ln φ versus ln *t* for rPET/rPP/PMDA blend at different PMDA content (e) 0 wt %, (f) 0.125 wt %, (g) 0.25 wt %, and (h) 0.375 wt %, (and (c) 0.5 wt %).

Mo’s kinetic method was applied to plot ln φ as a function of ln *t* at a given degree of crystallinity (Figure 6). For various polymer blends and composites, Mo’s theoretical prediction holds. As can be seen, correlation coefficients (>0.98) suggest that the experimental results and Mo’s method are consistent. The values of *F*(*T*) and α are summarized in Table 2.

**Table 2:** Kinetics Parameters^a^ for Non-isothermal Crystallization of rPET in Blends

				Δ*E*_a_ (kJ mol^–1^)		
sample	*X*_*t*_	*F*(*T*)	α	Kissinger	Takhor	Augis–Bennett
P1	30	4.07	1.69	316.49	312.45	308.39
	50	5.60	1.64			
	70	7.39	1.56			
	90	10.16	1.47			
P2	30	5.95	1.37	289.11	285.07	281.03
	50	7.48	1.34			
	70	9.16	1.31			
	90	12.13	1.22			
P3	30	7.85	1.11	284.71	280.69	276.65
	50	9.24	1.13			
	70	10.79	1.15			
	90	13.61	1.12			
P4	30	6.30	1.61	259.22	255.22	251.21
	50	8.45	1.53			
	70	10.61	1.53			
	90	14.30	1.45			
E1	30	4.95	2.45	268.87	264.83	260.78
	50	7.95	2.11			
	70	10.42	2.07			
	90	14.55	1.88			
E2	30	6.69	1.43	288.51	284.46	280.42
	50	8.35	1.40			
	70	10.14	1.37			
	90	13.36	1.27			
E3	30	7.78	1.07	297.15	293.11	289.07
	50	9.03	1.09			
	70	10.46	1.10			
	90	13.11	1.11			
E4	30	6.86	1.38	305.57	301.53	297.50
	50	8.47	1.36			
	70	10.26	1.32			
	90	13.36	1.22			
E5	30	7.07	1.34	297.16	293.13	289.09
	50	8.64	1.33			
	70	10.41	1.29			
	90	13.53	1.25			

a See Table 3 for sample compositions; *X*_*t*_: relative degree of crystallinity at time *t, F*(*T*): value of the cooling rate to reach a certain degree of crystallinity at unit crystallization time, α = *n*/*m* is the ratio of Avrami to Ozawa exponent, and Δ*E*_a_: activation energy of crystallization.

As seen, with the increase in *X*_*t*_ α changes slightly but *F*(*T*) increases, which indicates that at unit crystallization time, higher *X*_*t*_ requires a higher cooling rate. For all samples, α crystallization of rPET in blends varies in the range of 1–2.5 specifying that secondary crystallization growth occurs alongside primary crystallization during the non-isothermal crystallization. This is in good agreement with the results derived from Jeziorny modification to the Avrami model. Moreover, addition of rPP to formulations directed to higher α, which states the nucleating effect of the rPP phase. A higher amount of rPP in the blends led to a lower *F*(*T*), which points to a higher crystallization rate for the identical *X*_*t*_. Similar results were observed for the addition of PMDA.

**Figure 7:**
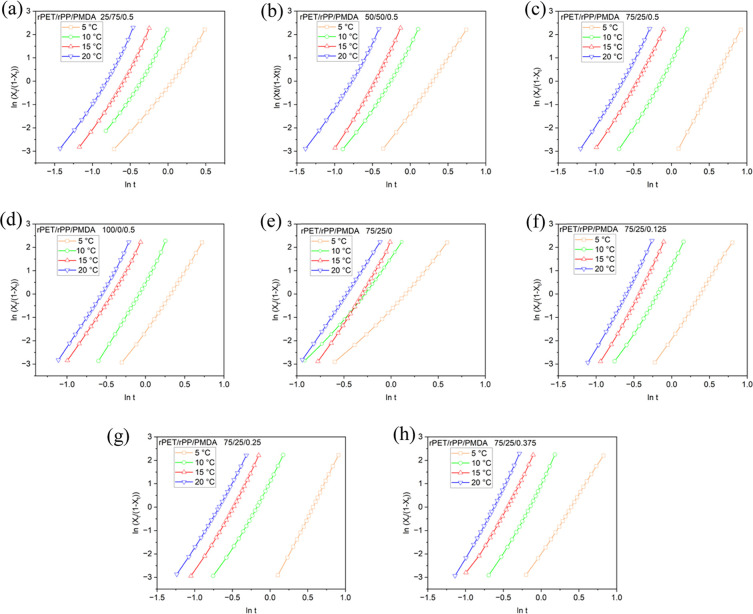
Plots of ln[*X_t_*/(1 – *X_t_*)] as a function of ln *t* (*X*_*t*_: relative degree of crystallinity at time *t*) for rPET/rPP/PMDA blends at various rPET/rPP content percentages of (a) 25/75, (b) 50/50, (c) 75/25, (d) 100/0, and % PMDA 0.5 wt. Plots of ln[*X_t_*/(1 – *X_t_*)] versus ln *t* for rPET/rPP 75/25 blends at different PMDA contents of (e) 0 wt %, (f) 0.125 wt %, (g) 0.25 wt %, and (h) 0.375 wt %, (and (c) 0.5 wt %).

Jeziorny-modified Avrami theory neglected the effects of the hindrance of crystallization caused by spherulites and secondary crystallization processes, which can be considered using Tobin theory (Figure 7). Calculating Tobin’s exponent (*n*_T_) and crystallization rate constant (*K*_T_) showed that the incorporation of rPP to blends results in higher *n*_T_ values in view of its heterogeneous nucleation effect, which implies faster completion of crystallization (or incomplete spherulites growth). Adding PMDA, decreased these values on the account of chain extension effect and branching on spherulite growth. *K*_T_ decreased with reducing rPP phase and increasing PMDA content in the blends. The results are consistent with Jeziorny-modified Avrami and Mo models.

**Figure 8:**
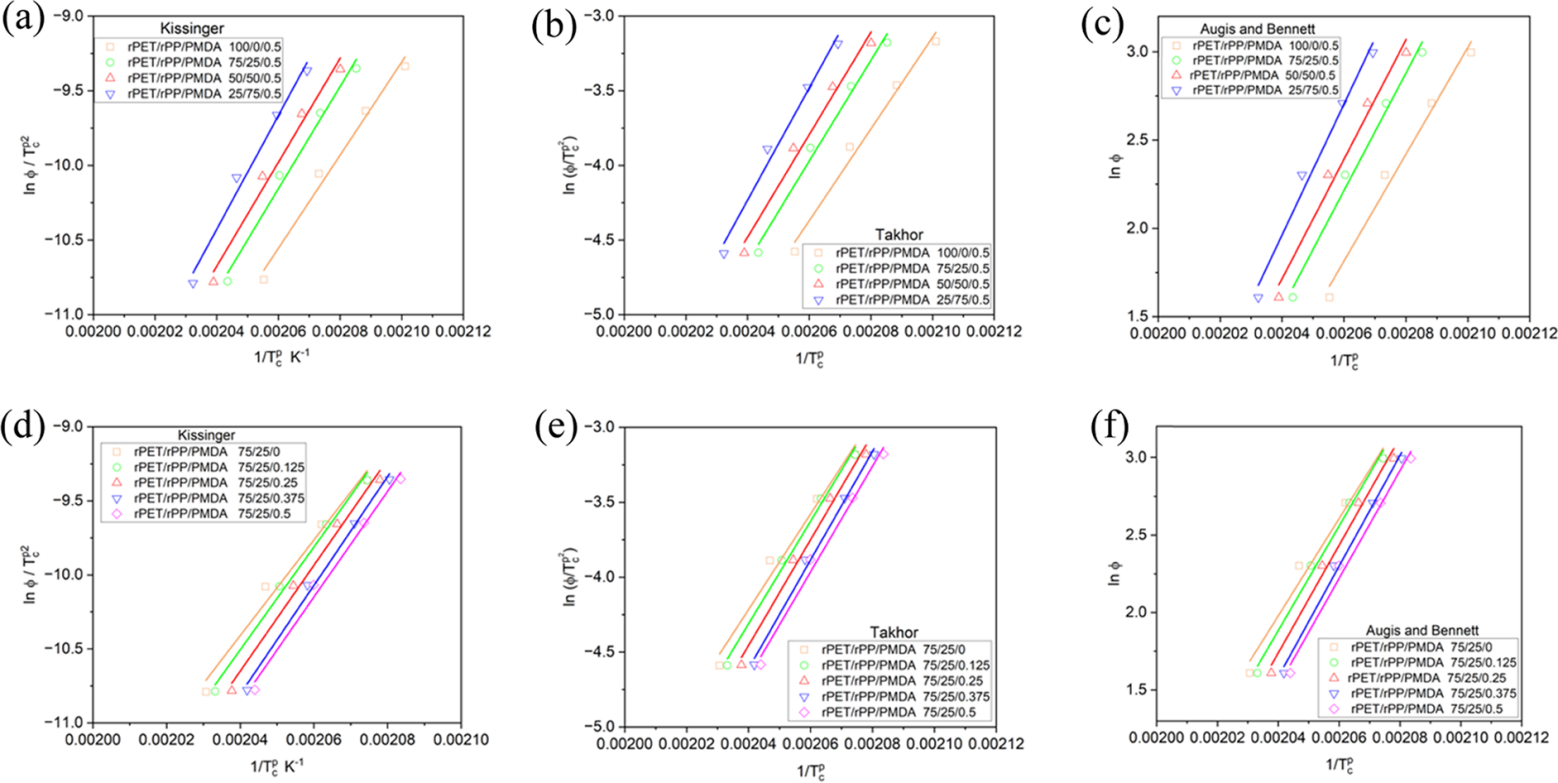
(a) Kissinger, (b) Takhor, and (c) Augis and Bennett plots for the required activation energy for the crystallization of rPET at different content of rPP. (d) Kissinger, (e) Takhor, and (f) Augis and Bennett plots for required activation energy for the crystallization of rPET at different PMDA contents.

Activation energy (Δ*E*_a_) of the non-isothermal crystallization process was derived based on Kissinger, Takhor, and Augis–Bennett models (Table 4). Δ*E*_a_ was obtained from the slope of plots in Figure 8, which is summarized in Table 2. Expectedly, Δ*E*_a_ increased with increasing PMDA, due to its restriction effects on rPET chain mobility. Similar results were reported elsewhere for PP/rPET^47^ and PET/clay nanocomposite.^48^ Nucleation and restriction effects affect rPET crystallization simultaneously. While the former speeds up crystallization, the latter exhibits hindrance effects. Although rPP promotes nucleation, its restriction effects on crystallite growth lead to a higher Δ*E*_a_, which indicates that the latter factor dominates. Increasing PMDA content, caused Δ*E*_a_ to increase, which specifies that LCBs restrict crystallization.

**Figure 9:**
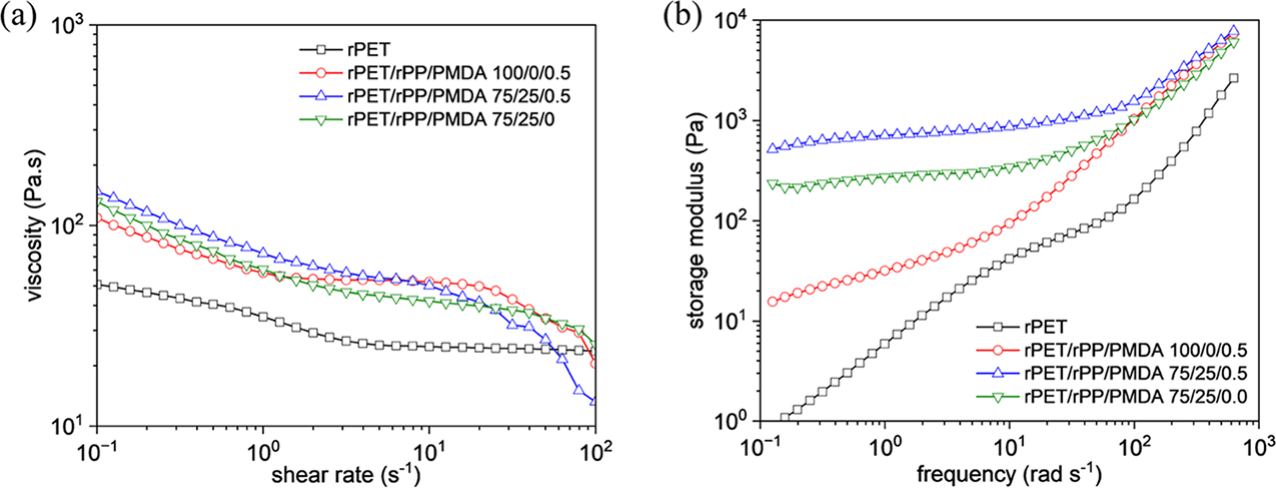
(a) Viscosity versus shear rate, and (b) storage modulus as a function of frequency at 265 °C for rPET/rPP/PMDA blends (samples P4, E5, and E1) in comparison with the neat rPET.

The melt viscosity as a function of shear rate (γ̇), and storage modulus versus frequency for rPET/rPP/PMDA blends (samples P4, E5, and E1 in comparison with the neat rPET) are shown in Figure 9. As can be seen, the incorporation of the rPP and PMDA into the rPET matrix elevates melt viscosity at low shear rates and changes the viscoelasticity of the blend system. As can be seen, PMDA increases the viscosity at a low γ̇ regime, which is attributed to the extension and branching of PET chains, and induce shear thinning at higher γ̇. An increase in melt viscosity and shear thinning behavior at higher γ̇ due to chains disentanglement is relevant for industrial processing.^49^Figure 9b shows that after incorporation of PMDA (sample P4) and rPP/PMDA (samples E5 and E1) into the rPET matrix, the storage modulus is remarkably increased up to one and more than two orders of magnitude. This can be interpreted with the formation of LCBs in the PET matrix, whose effect becomes more pronounced after the incorporation of rPP.

**Figure 10:**
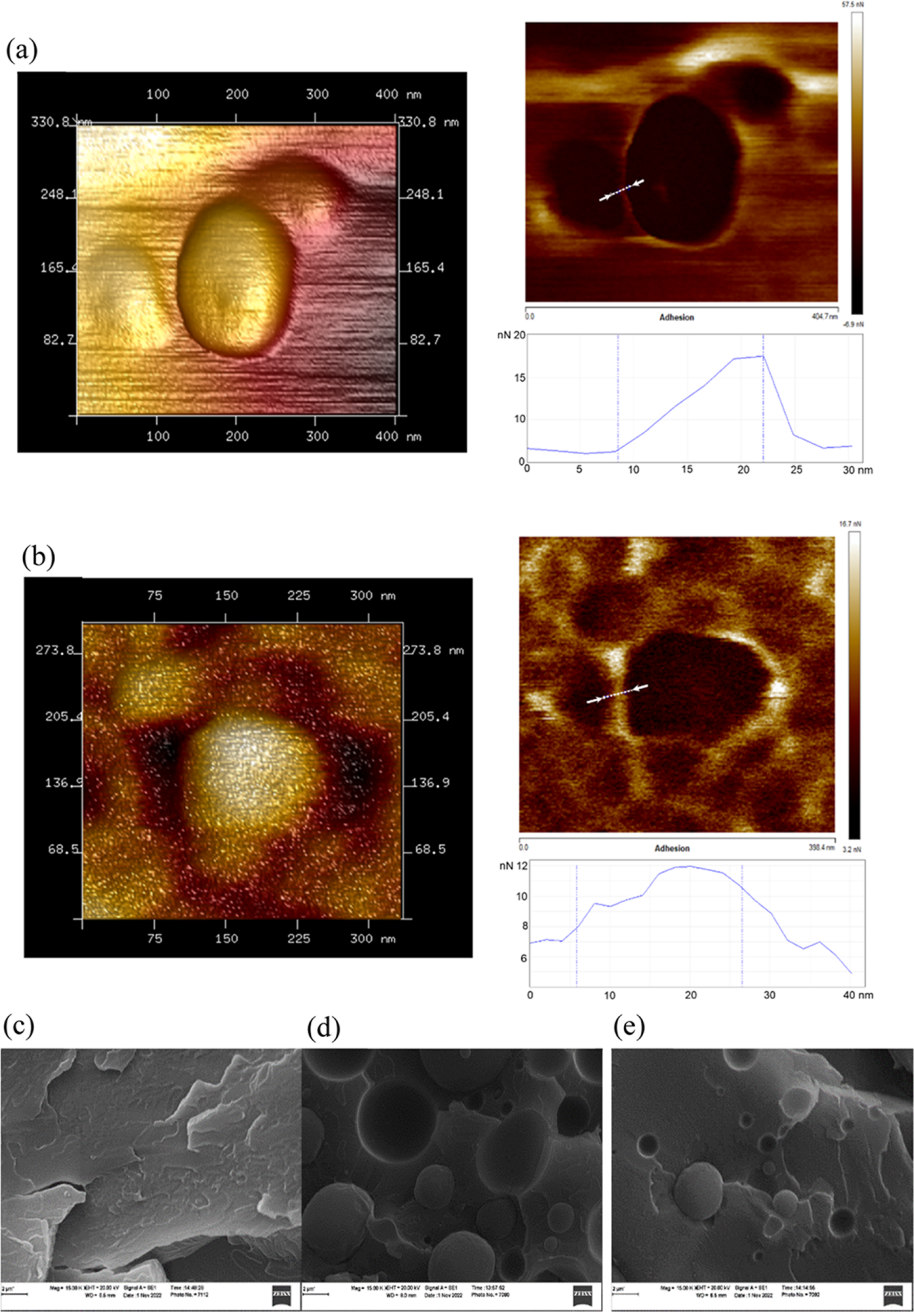
Atomic force microscopy images of (a) rPET/rPP (sample E1), and (b) rPET/rPP/PMDA (sample E5) blends. The left image displays topographic and the panel demonstrates the adhesion map across the interfacial regions between the rPET (matrix) and rPP (dispersed phase). Scanning electron microscopy images of (c) rPET, (d) rPET/rPP (sample E1), and (e) rPET/rPP/PMDA (sample E5) (scale bar 2 μm).

Figure 10a,b displays atomic force micrographs of interfacial domains in rPET/rPP and rPET/rPP/PMDA blends. As can be seen, the incorporation of PMDA induced interphase diffusion and adhesion between the dispersed phase (rPP) and the rPET matrix. This effect can be described in terms of increased matrix melt viscosity, which improves mixing in the course of reactive blending, and eventually resulted in broadening of the interfacial domains. This explanation was verified based on morphological analysis using scanning electron microscopy. As displayed in Figure 10d,e, the size of the dispersed phase becomes smaller after incorporation of the chain extender agent into the blend. In fact, using PMDA reduces interfacial tension and improves the adhesion between dispersed rPP droplets in the rPET matrix, which eventually resulted in uniformity of rPP phase boundaries.

## 3 Experimental Section

### 3.1 Materials and Compounding

Recycled poly(ethylene terephthalate) (rPET, grade 3000) derived from diverse feedstocks of post-use containers was supplied by Ex-Tech Plastics. Recycled polypropylene (rPP) (grade 308A) was supplied by KW Plastics. Pyromellitic dianhydride was purchased from Sigma-Aldrich. Prior to compounding, rPET flakes were dried at 100 °C for 24 h in a vacuum oven to minimize hydrolytic degradation. To reduce thermo-oxidative degradation, Irganox 1010 supplied by BASF was used as an antioxidant stabilizer. The reactive melt compounding process was performed using an internal mixer (IntelliTorque Plasticorder, Brabender CWB) at 265 °C, and 90 rpm for 10 min. The composition of samples comprising rPET flakes, rPP granules, and PMDA are summarized in Table 3.

**Table 3:** Formulation of rPET/rPP/PMDA Compounds

sample code	rPET (wt %)	rPP (wt %)	PMDA (wt %)
P1	25	75	0.5
P2	50	50	0.5
P3	75	25	0.5
P4	100	0	0.5
E1	75	25	0
E2	75	25	0.125
E3	75	25	0.25
E4	75	25	0.375
E5	75	25	0.5

### 3.2 Characterization Methods

#### 3.2.1 Differential Scanning Calorimetry

Non-isothermal crystallization kinetics of rPET/rPP/PMDA blends was analyzed by differential scanning calorimetry (DSC). DSC measurements were performed using a DSC 250 (TA Instruments). The DSC equipment was continuously purged with nitrogen gas at a flow rate of 50 mL min^–1^. 5–10 mg of samples were initially heated from ambient temperature to 280 °C at a heating rate of 10 °C min^–1^ and held at 280 °C for 2 min to remove thermomechanical history. Subsequently, the samples were cooled at varying rates of 5, 10, 15, and 20 °C min^–1^. Eventually, the second heating runs were carried out.

#### 3.2.2 Fourier Transform Infrared Analysis

Initially, polymeric samples were hot-pressed at 265 °C into sheets with 500 μm thickness using a Carver bench-top press and then analyzed via Fourier transform infrared (FTIR) spectroscopy (Spectrum Two FTIR spectrometer, PerkinElmer). The scan number was 32, and the resolution was 4 cm^–1^.

#### 3.2.3 Rheological Characterization

Rheological measurements were implemented by means of a Discovery Hybrid rheometer (HR 30, TA Instruments) using parallel plates (diameter 50 mm, gap 1 mm) at 265 °C. Initially, the samples were compression molded into disks with a thickness of 1 mm. Melt viscosity was measured at a shear rate of 0.1–100 s^–1^. Oscillatory frequency sweep measurements were conducted in the linear viscoelasticity region determined based on strain sweep experiments.

#### 3.2.4 Atomic Force Microscopy

A Bruker Dimension Icon atomic force microscope (AFM, Bruker Nano Inc.) was employed in this work. The peak force quantitative nanomechanical mapping (PF-QNM) mode was used to monitor the nanostructure and interface of rPET and rPP domains. RTESPA-300-30 probes were selected for the analysis. The nominal probe spring constant was 40 N/m, and the radius of the tip was 30 nm. The spring constant of each probe was pre-calibrated by the manufacturer. The deflection and amplitude sensitivities of the probes were calibrated by the contact method with a sapphire sample before analysis. In this study, the adhesion force was recorded.^50^ The adhesion force was used to probe rPET/rPP interfacial domains and study the effect of PMDA.

#### 3.2.5 Scanning Electron Microscopy

Initially, samples were mounted onto aluminum support stubs with a piece of double-stick carbon tape and then coated with gold using an EMS 550 Auto Sputter Coating Device (Electron Microscopy Sciences). Samples were then analyzed with a Zeiss EVO 50 Variable Pressure SEM (Carl Zeiss SMT, Inc.) operated at 20 kV, and micrographs were captured at 15K magnification.

#### 3.2.6 Theoretical Approach to Non-Isothermal Crystallization Kinetics

The mathematical models used in this work are summarized in Table 4.^51−57^ Avrami equation with general expression of 1 – *X*_*t*_ = e^(−*kt*^*n*^^), where *X*_*t*_ is the crystallinity fraction at time *t*, *k* is the growth rate, and *n* represents the nucleation mechanism and growth dimension, is commonly used to analyze crystallization rate under isothermal conditions.^58^ Several methods have been developed to investigate non-isothermal crystallization parameters, and many of these formulations are based on the Avrami equation.

**Table 4:** Models Used to Study Non-Isothermal Crystallization Kinetics of rPET/rPP/PMDA Blends

methods	mathematical models	plots	ref
Jeziorny extended Avrami	ln[−ln(1 – *X_t_*)] = ln Z_c_ + *n* ln *t*	ln[−ln(1 – *X_t_*)] vs ln *t*	(51)
	ln *Z*_c_ = ln *Z_t_*φ^–1^		
	*t* = (*T*_c_^on^ – *T*)/φ		


*Z*_*t*_ and *Z*_c_: Avrami and Jeziorny extended Avrami rate constant, *n*: Avrami exponent, φ: cooling rate			
Ozawa	ln[−ln(1 – *X*_*t*_)] = ln *K*(*T*) – *m *ln φ	ln[−ln(1 – *X_t_*)] vs ln φ	(52)
*K*(*T*): constant related to the overall crystallization rate, *m*: Ozawa index			
Mo	ln φ = ln *F*(*T*) – α ln *t*	ln φ vs ln *t*	(53)

α = *n*/*m* is the ratio of Avrami to Ozawa exponent, *F*(*T*): value of the cooling rate to reach a certain degree of crystallinity at unit crystallization time			
Tobin			(54)
*n*_T_: Tobin exponent, *K*_T_: Tobin rate constant			
Kissinger			(55)
*T*_*c*_^*p*^: crystallization peak temperature, Δ*E*_a_: activation energy of crystallization, *R*: universal gas constant			
Takhor			(56)
Augis and Bennett			(57)

## 4 Conclusions

Non-isothermal crystallization of the recycled PET phase in blends with recycled PP compounded in the presence of PMDA as a chain extender was studied. It was found that with increasing rPP content in blends, the crystallization temperature of the rPET phase increases. Increasing PMDA content in blends at a given cooling rate lowered the crystallization peak temperature. This effect was attributed to the introduction of long chain branches through chemical reactions among PMDA anhydride and rPET terminal groups, which was verified by FTIR spectroscopy. The kinetics of non-isothermal crystallization of blends was studied using four kinetics models summarized in Table 4. Jeziorny-modified Avrami (*n*) and Tobin (*n*_T_) values indicate that PMDA inclusions promote rPET spherulite formation toward unsophisticated geometries, improving crystallization rates. Increasing rPP in blend compositions decreases *F*(*T*), indicating faster crystallization. As a result of the restriction effect on rPET chain mobility, the activation energy of crystallization was found to increase with increasing rPP and PMDA content. Nanomechanical mapping demonstrated the broadening of rPET/rPP interfacial domains in view of chain extension and branching effect of PMDA, which was supported by increased grain boundaries in scanning electron micrographs. Rheological measurements revealed a remarkable increase in melt viscosity and elasticity of blends compared to neat rPET, which is relevant from the processability perspective.
